# MicroRNA Maturation and MicroRNA Target Gene Expression Regulation Are Severely Disrupted in Soybean *dicer-like1* Double Mutants

**DOI:** 10.1534/g3.115.022137

**Published:** 2015-12-15

**Authors:** Shaun J. Curtin, Jean-Michel Michno, Benjamin W. Campbell, Javier Gil-Humanes, Sandra M. Mathioni, Reza Hammond, Juan J. Gutierrez-Gonzalez, Ryan C. Donohue, Michael B. Kantar, Andrew L. Eamens, Blake C. Meyers, Daniel F. Voytas, Robert M. Stupar

**Affiliations:** * Department of Plant Pathology, University of Minnesota, St. Paul, Minnesota, 55108; † Department of Agronomy and Plant Genetics, University of Minnesota, St. Paul, Minnesota, 55108; ‡ Department of Genetics, Cell Biology and Development, University of Minnesota, Minneapolis, Minnesota 55455; § Center for Genome Engineering, University of Minnesota, Minneapolis, Minnesota 55455; ** Department of Plant and Soil Sciences, University of Delaware, Newark, Delaware 19711; †† School of Environmental and Life Sciences, The University of Newcastle, Callaghan, New South Wales 2308, Australia

**Keywords:** dicer-like, miRNA, genome engineering, ZFN, soybean

## Abstract

Small nonprotein-coding microRNAs (miRNAs) are present in most eukaryotes and are central effectors of RNA silencing-mediated mechanisms for gene expression regulation. In plants, DICER-LIKE1 (DCL1) is the founding member of a highly conserved family of RNase III-like endonucleases that function as core machinery proteins to process hairpin-like precursor transcripts into mature miRNAs, small regulatory RNAs, 21–22 nucleotides in length. Zinc finger nucleases (ZFNs) were used to generate single and double-mutants of putative soybean *DCL1* homologs, *DCL1a* and *DCL1b*, to confirm their functional role(s) in the soybean miRNA pathway. Neither DCL1 single mutant, *dcl1a* or *dcl1b* plants, exhibited a pronounced morphological or molecular phenotype. However, the *dcl1a*/*dcl1b* double mutant expressed a strong morphological phenotype, characterized by reduced seed size and aborted seedling development, in addition to defective miRNA precursor transcript processing efficiency and deregulated miRNA target gene expression. Together, these findings indicate that the two soybean *DCL1* paralogs, *DCL1a* and *DCL1b*, largely play functionally redundant roles in the miRNA pathway and are essential for normal plant development.

Plants produce two main small RNA (sRNA) populations, microRNAs (miRNAs) and small-interfering RNAs (siRNAs) (Chen 2010). miRNAs are generated from hairpin-like precursor transcripts that are partially self-complementary; the 21–22 nucleotide (nt) mature miRNAs are crucial for directing the targeting of endogenous messenger RNA (mRNA) transcripts for RNA-induced silencing complex (RISC)-mediated cleavage or, in some cases, translational repression (Millar and Waterhouse 2005; Bartel 2009; Voinnet 2009). siRNAs, which represent approximately 90–95% of the global sRNA population in plants, are generated from perfectly double-stranded RNA (dsRNA) derived from replicating viruses, transposons and other classes of repetitive DNA, or introduced transgenes (Waterhouse *et al.* 1998; Baulcombe 2004; Voinnet 2009). In addition, secondary siRNAs (21–22 nt) are derived from either mRNAs of protein-coding genes, or from long nonprotein-coding RNAs. The enzyme family responsible for processing a sRNA from its precursor transcript is the Dicer (Dcr) family of RNase III-like endonucleases (Bernstein *et al.* 2001). Many eukaryotes encode multiple Dcr family members, and each Dcr can mediate specialized, yet often redundant roles. In *Caenorhabditis elegans*, however, a single Dcr is responsible for both miRNA and siRNA production (Duchaine *et al.* 2006). *Drosophila melanogaster* encodes two Dcrs: Dcr1 is required for miRNA biogenesis and Dcr2 is responsible for siRNA production (Tomari and Zamore 2005). The first plant Dcr identified was in *Arabidopsis thaliana* (*Arabidopsis*) via the characterization of a mutant plant originally termed *carpel factory* (*caf*), due to the floral meristem defects expressed by *caf* mutants (Jacobsen *et al.* 1999). Subsequently, the *CAF* gene was determined to encode a protein with similarities to animal Dcrs, and was therefore renamed *DICER-LIKE1* (*DCL1*). Furthermore, the severe developmental phenotype expressed by *caf* plants has been shown to result from the ectopic expression of genes that are normally under miRNA regulation. In brief, the *dcl1* mutation led to reduced miRNA precursor transcript processing (cleavage) efficiency, reduced mature miRNA accumulation, and deregulated miRNA target transcript expression.

To date, several *Arabidopsis dcl1* alleles have been reported, including the weak *dcl1-7* (*short integument1-1*) hypomorphic mutant and the embryo lethal *dcl1-5* (*suspensor1-5*) mutant (Schauer *et al.* 2002). In rice, hairpin directed *DCL1* knock-down transformants express severe developmental phenotypes in strongly silenced lines and lesser pleiotropic developmental defects in weakly silenced lines (Liu *et al.* 2005). In common bean, *Phaseolus vulgaris*, the authors demonstrated disruption of miR399 expression in composite plants transformed with a PvDCL1 hairpin reagent (Valdes-Lopez *et al.* 2008). A maize *dcl1* mutant plant named *fuzzy tassel* (*fzt*) was recently identified by screening an ethyl methane sulfonate-mutagenized population and *fzt* plants displayed severe developmental defects (Thompson *et al.* 2014). The model moss species, *Physcomitrella patens* encodes four DCL proteins, including two DCL1 homologs, DCL1a and DCL1b. Curiously, PpDCL1a and PpDCL1b have been demonstrated to play distinct functional roles in the *P. patens* miRNA pathway (Khraiwesh *et al.* 2010). The PpDCL1a protein functions identically to its *Arabidopsis* ortholog AtDCL1, processing miRNA precursor transcripts as well as being involved in the production of the phased class of siRNAs, termed pasiRNAs. However, the PpDCL1a paralog, PpDCL1b, is not required for miRNA production. Instead, PpDCL1b is involved in the miRNA-directed target transcript cleavage stage of the *P. patens* miRNA pathway (Khraiwesh *et al.* 2010).

The genome of the paleopolyploid legume *Glycine max* (soybean) has retained a high level of gene duplication, with approximately 75% of the genes present in more than a single copy (Schmutz *et al.* 2010; Roulin *et al.* 2013). This identifies soybean as an interesting system for investigating the divergence of ancient homologous genes, with the potential to exhibit patterns of subfunctionalization or neofunctionalization (Force *et al.* 1999; Curtin *et al.* 2012). Soybean has retained two putatively functional *DCL1* gene copies that encode proteins which are highly conserved orthologs of the well-characterized AtDCL1. The Gm*DCL1a* and Gm*DCL1b* loci are located in a large syntenous block between chromosomes 3 and 19 (Schmutz *et al.* 2010; Curtin *et al.* 2012). Gm*DCL1a* was designated with the gene identifier Glyma03g42290 in the version 1 genome assembly (Schmutz *et al.* 2010) and Glyma.03g262100 in the version 2 assembly. Gm*DCL1b* was designated the identifiers Glyma19g45060 and Glyma.19g261200 in the first and second genome assemblies, respectively (Schmutz *et al.* 2010). While some transcriptional differences have been noted for the two Gm*DCL1* gene copies (Curtin *et al.* 2012), the duplicated nature of the two soybean *DCL1* genes makes it difficult to define the functional role each plays in miRNA-directed RNA silencing in soybean.

While a number of soybean mutant lines have been generated via random mutagenesis (Cooper *et al.* 2008; Hancock *et al.* 2011; Cui *et al.* 2013; Bolon *et al.* 2014), mutants for these two Gm*DCL1* loci are not readily available. Therefore, targeted mutagenesis using site-specific nucleases (SSNs) offers a promising approach for creating site-specific mutations in these duplicate genes, as has been previously demonstrated in soybean (Curtin *et al.* 2011; Haun *et al.* 2014; Jacobs *et al.* 2015; Sun *et al.* 2015). SSNs are synthetic enzymes with programmable DNA target specificities that are used to introduce double-strand breaks at specific loci. The double-stranded break is rapidly repaired by the host’s error-prone nonhomologous end joining (NHEJ) repair pathway, a repair mechanism that often results in the introduction of small insertion-deletions (indels) that range in size from one to many hundreds of base pairs (Voytas 2013). In a previous study, we engineered a zinc finger nuclease (ZFN) to target both Gm*DCL1* copies and demonstrated its capacity to generate mutations in somatic hairy-root tissue. Here, the *DCL1*-targeting ZFN was used to generate heritable *dcl1a* and *dcl1b* mutations in whole soybean plants. Furthermore, the mutations were combined via a standard genetic crossing approach to generate the *dcl1a/dcl1b* double mutant. The miRNA pathway was assessed in single and double mutant plants to gain new insights into the functional redundancies exhibited by the Gm*DCL1* paralogs in this gene expression regulatory pathway that is central to plant development.

## Material and Methods

### The transformation of zinc finger nucleases targeting DCL1

The identification of the *DCL1* target site, construction of the *DCL1* zinc finger nuclease, and functional confirmation in somatic soybean tissue for targeting both copies of the *DCL1a* and *DCL1b* nuclease has been previously reported (Curtin *et al.* 2011). The ZFN transgene was transformed into *Agrobacterium rhizogenes* strain 18r12, and the DCL1-ZFN harboring *A. rhizogenes* culture was used to transform whole plant soybean cultivar ‘Bert’ as described previously (Paz *et al.* 2006; Curtin *et al.* 2011).

### Detection of ZFN-induced mutations

DNA was extracted from soybean leaf chads of ZFN-transformed plants using the previously described CTAB extraction method (Curtin *et al.* 2008). To detect ZFN-induced mutations, both the *DCL1a* and *DCL1b* targeted loci were amplified using primers; 5′-ATCTGTACTAATGCAGAGGATCTGG-3′ and 5′-CAAGTGATCCAGG CAGTGGGTGTACG-3′ for *DCL1a*, and 5′-ATCCAGCCAAGCCTTCCGTATCCAC-3′ and 5′-AAGTTTTGTCATGTGTCTCTTCG-3′ for *DCL1b*. The resulting amplicons were digested for 6 hr at 37° with *Psi*I and visualized via standard gel electrophoresis. Cleavage-resistant amplicons were gel purified, cloned into the pGEM-T Easy vector (Promega), and sequenced. Subsequent genotyping of the *DCL1* loci in nontransgenic progeny was performed using the PCR amplicon digestion strategy previously described in Curtin *et al.* (2011).

### Development of single and double mutants and phenotypic analyses

T_0_ plants harboring mutations were allowed to self-pollinate to homozygous specific mutated alleles and remove the ZFN transgenes through genetic segregation in subsequent generations. Different combinations of alleles at the *DCL1a* and *DCL1b* loci were stacked together through a series of cross-fertilization and selfing generations. Plants were genotyped for the introduced transgene using PCR and genotyped for the mutant alleles using PCR followed by restriction digestion assays. The single and double-mutants were monitored for gross morphological phenotypes compared to the fully wild-type segregants. For the single mutant phenotypic analyses, seeds were germinated in sterile Metro Mix, inoculated with *Bradyrhizobium* USDA110 strain, and cultivated in a temperature controlled growth chamber for 14 d.

### Small RNA and RNA-Seq library sequencing and informatics analyses

Total RNA from plant leaves or whole-plants was isolated using the Purelink Plant RNA Reagent (Invitrogen; Cat#12322-012). Small RNA libraries were constructed using the Illumina TruSeq Small RNA sample preparation kit (RS-200-0012) and RNA-Seq libraries were prepared using the Illumina TruSeq RNA sample preparation kit (RS-122-2001). All libraries were sequenced on an Illumina HiSeq2000 instrument at the Delaware Biotechnology Institute of the University of Delaware. The raw sequences were processed by bioinformatic removal of linker adaptor sequences and the trimmed reads matched to the soybean genome, Wm82.a2.v1 (Schmutz *et al.* 2010). To facilitate library comparison between each assessed mutant background, the count/abundance of each mutant population was normalized to its corresponding control based on a sequencing read depth and visualized using the gplots package in R statistical environment (R Development Core Team 2013). Raw RNA-Seq libraries were processed using *Tophat* (Trapnell *et al.* 2009) to map the reads to the soybean genome, Wm82.a2.v1 (Schmutz *et al.* 2010). Genome mapped reads were assembled and a ‘merged’ transcriptome assembly was generated using *Cufflinks*. Quantification of gene expression levels was done using *featureCounts* (Liao *et al.* 2014) to create count tables. These count tables were imported to R statistical environment (R Development Core Team 2013) where lowly expressed reads were filtered out and the data were normalized using *DESeq* to identify differentially expressed genes. Due to the difficulty of generating replicates, we needed to calculate dispersion under the assumption that the mean of the libraries being compared would be a good predictor for the dispersion. Under this assumption, it is possible to calculate differentially expressed transcripts from the samples (Love *et al.* 2014). While several miRNA targets were previously identified (Arikit *et al.* 2014), their analysis was done on a previous version of the soybean genome. In order to identify differentially expressed miRNA target genes with the current genome annotation, it was necessary to predict and validate individual miRNA target genes. The identification of miRNA targets was performed using the *sPARTA* package (Kakrana *et al.* 2014) built-in target prediction module *mirFerno*. Nine PARE libraries were utilized to validate those targets and targets were determined ‘real’ if the target had an adjusted *P*-value <0.05. The validated targets were then mapped back to the previously identified differentially expressed transcripts to confirm differentially expressed miRNA target genes.

### Quantitative real time PCR (RT-qPCR)

RT-qPCR was used to quantify the relative expression of protein-encoding transcripts determined to be miRNA target genes. Reactions were performed using FastStart Universal SYBR Green Mix kit (Roche) on a LightCycler 480 Instrument (Roche). Primers were designed for a small subset of miRNA target transcripts based on previously published reports (Kulcheski *et al.* 2011; Song *et al.* 2011). *ACTIN* (gene model Glyma.15G050200) and *ELONGATION FACTOR1 ALPHAGENE* (Glyma.17G186600) were used as reference genes to normalize miRNA target gene expression. RT-qPCR reactions consisted of 0.3 μM of each primer and 1× FastStart Universal SYBR Green Mix in a final reaction volume of 10 μl with the cDNA template obtained from 2000 ng of total RNA. Primer efficiencies and Cq values were determined using the LingRegPCR v2013.0 software (Ruijter *et al.* 2009). Normalized copy number and expression were calculated using previously-described equations (Hellemans *et al.* 2007) for multiple reference genes, and the results were standardized using an adapted version of the Microsoft Excel Qgene template (Muller *et al.* 2002). Three technical replicates were performed for each of the two biological replicates. Error bars represent standard error of the mean between biological replicates.

### Data availability

RNA-Seq raw data are available on Sequence Read Archive under the accession GSE76036 or can be accessed through the soybean gene analysis link (https://mpss.udel.edu/dbs/index.php?SITE=soy_RNAseq).

## Results

### Targeted mutagenesis of DCL1a and DCL1b with zinc finger nucleases

**Figure 1:**
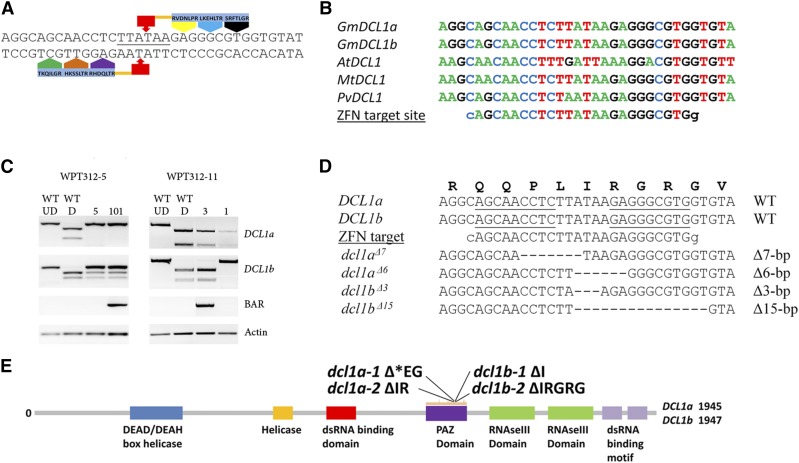
Generation of whole plant *dcl1* mutants in soybean using zinc finger nuclease. (A) Schematic of zinc finger nuclease (ZFN) monomers bound to the target DNA. The target sequence of both left and right zinc finger arrays (ZFAs) recognize a 9 bp sequence, with underlined sequences representing the *Psi*I restriction site used in conjunction with a PCR-based assay to identify putative transformants harboring the desired mutations. (B) Sequence alignment of the homologous *DCL1*a and *DCL1*b ZFN target sites between legume species and *Arabidopsis*, highlighting the high level of sequence conservation of the targeted region. (C) The PCR digestion assays of T_1_ progeny from putative transformant lines, WPT312-5 and WPT312-11. Undigested bands indicate mutated DNA sequences. The presence of the ZFN transgene was detected by an additional PCR screen specific for the *BAR* transgene. ‘D’ and ‘UD’ denotes digested and undigested amplicons, respectively. (D) Sequence confirmation of the *dcl1a* and *dcl1b* mutant alleles identified in ZFN-transformed whole-plants. (E) A schematic of the DCL1 protein, highlighting the location of the four mutations within the exon encoding the functionally essential PAZ domain.

Previous work investigating the relative transcription of *DCL1a* and *DCL1b* across a panel of stress treatments indicated only minor expression differences for these two genes (Curtin *et al.* 2012). Publicly available RNA-Seq data from various soybean tissues (Nakano *et al.* 2006; Severin *et al.* 2010) further confirmed that *DCL1a* and *DCL1b* transcript levels are relatively similar to one another with no obvious signs of transcriptional specialization. Mutations for these genes were therefore desirable to investigate potential divergences between the *DCL1* gene copies. Having previously reported a highly active CoDA-engineered ZFN (Sander *et al.* 2011) that can introduce somatic mutations in *DCL1a* and *DCL1b* (Curtin *et al.* 2011), we sought to introduce these reagents into whole plants with the purpose of creating knock-out mutations in both *DCL1* loci (Figure 1A). Since the targeted region is located in the seventeenth and eighteenth exon of *DCL1a* and *DCL1b* respectively, coding sequences that encode for the functionally crucial PAZ domain, it was presumed that frameshift mutations at the targeted site would result in the generation of the desired functional knock-outs. Furthermore, the ZFN target site is highly conserved across multiple plant species, including *Arabidopsis*, Medicago and common bean (Figure 1B), further suggesting that the introduction of in-frame mutations in this region would perturb normal gene function.

**Figure 2:**
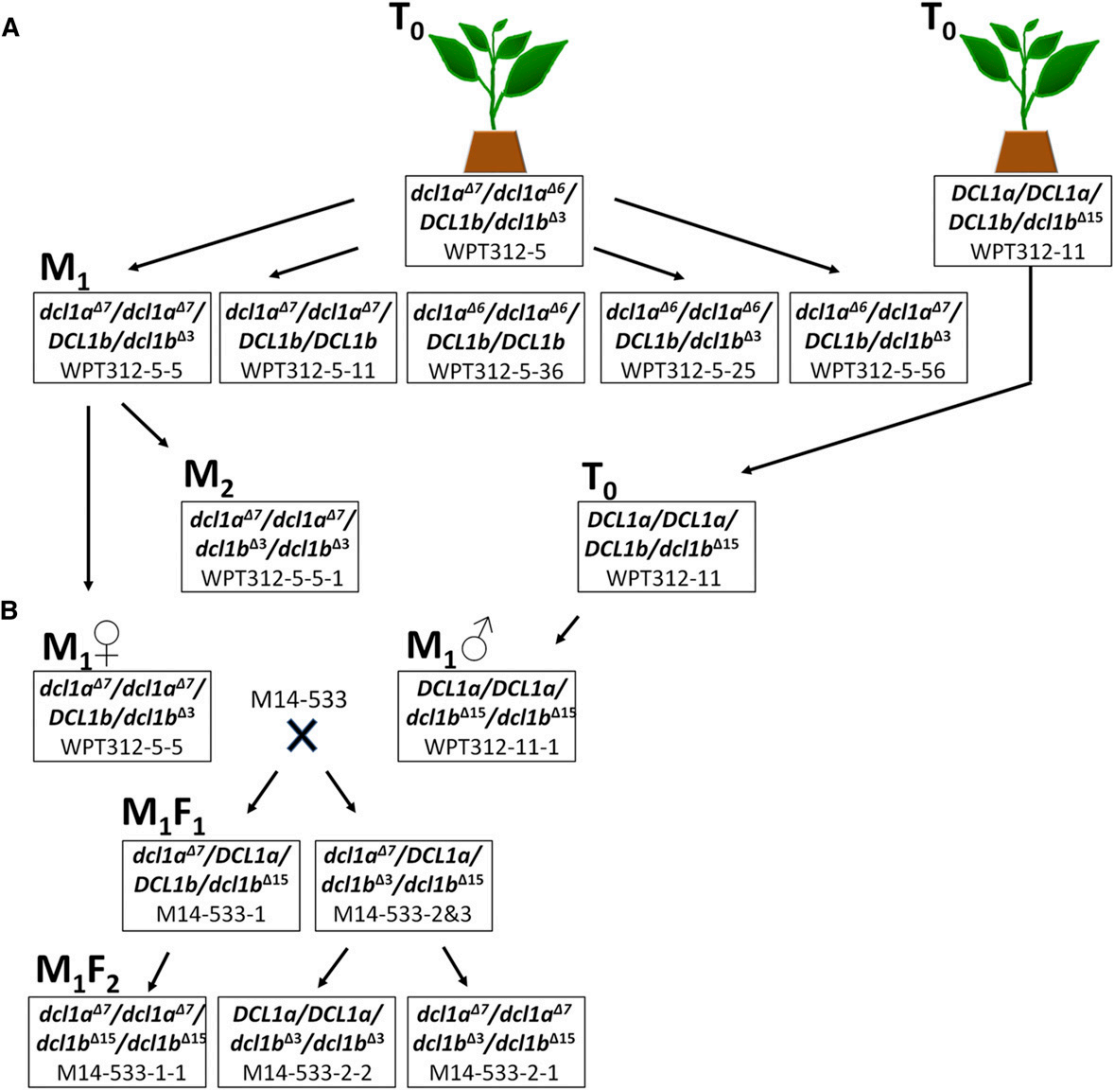
Schematic outlining the experimental approach used to generate single and double *dcl1* mutants. (A) Single and double *dcl1* mutations were identified from whole plant transformants, WPT312-5 and WPT312-11. The two T_0_ plants were self-fertilized, and progeny screened for both single and double mutations (among the *dcl1a*^Δ^*^7^*, *dcl1a*^Δ^*^6^*, *dcl1b*^Δ^*^15^* alleles) as well as removal of the ZFN (zinc finger nuclease) transgenes by genetic segregation. (B) The WPT312-5-5 (*dcl1a*^Δ^*^7^/dcl1a*^Δ^*^7^/DCL1b/dcl1b*^Δ^*^3^*) and WPT312-11-1 (*DCL1a/DCL1a/dcl1b*^Δ^*^15^/dcl1b*^Δ^*^15^*) plants were used as parents for a cross-fertilization experiment to introduce the *dcl1b*^Δ^*^15^* mutant allele into the *dcl1a*^Δ^*^7^/dcl1a*^Δ^*^7^/DCL1b/DCL1b* background, and to also isolate the *dcl1b*^Δ^*^3^* mutant allele in a wild-type *DCL1a* background.

Two soybean T_0_ plants, WPT312-5 and WPT312-11, determined to putatively harbor site-specific mutations, were recovered from a pool of 12 transformant lines (Supporting Information, File S1). Respective 6 bp and 7 bp deletions were identified at the two *DCL1a* homologs in the T_0_ plant WPT312-5. This plant was also found to have a mono-allelic 3 bp deletion of one *DCL1b* locus. Therefore, the genotype of the T_0_ plant WPT312-5 was denoted as *dcl1a*^Δ^*^6^/dcl1a*^Δ^*^7^/DCL1b/dcl1b*^Δ^*^3^* (Figure 2). Mutations were also confirmed in the second T_0_ plant, WPT312-11, which harbored a mono-allelic 15 bp deletion at the *DCL1b* locus (genotype was denoted as *DCL1a/DCL1a/DCL1b/dcl1b*^Δ^*^15^*) (File S1). Both plants were self-fertilized and the resulting T_1_ progeny screened to confirm heritable transmission of the mutated alleles, along with the removal of one or more transgenes by genetic segregation (Figure 1C). Six progeny were identified that carried the introduced mutations, but did not carry the transgenes. Since these mutant segregants were nontransgenic, this was considered the M_1_ generation (Figure 2). Seed from these M_1_ plants were bulk harvested for further analyses. The names and mutant allele composition of these plants are displayed in Table 1. The mutated alleles in the M_1_ plants were sequenced and heritable transmission of each mutation was confirmed (Figure 1, D and E, and File S2).

**Table 1:** Combinations of *dcl1a* and *dcl1b* mutant alleles

Plant Identifier	Genotype	Generation	Transgene
WPT312-5	*dcl1a*^Δ^*^7^/dcl1a*^Δ^*^6^/DCL1b/dcl1b*^Δ^*^3^*	T_0_	Yes
WPT312-11	*DCL1a/DCL1a/DCL1b/dcl1b*^Δ^*^15^*	T_0_	Yes
WPT312-5-11	*dcl1a*^Δ^*^7^/dcl1a*^Δ^*^7^/DCL1b/DCL1b*	M_1_	None
WPT312-5-36	*dcl1a*^Δ^*^6^/dcl1a*^Δ^*^6^/DCL1b/DCL1b*	M_1_	None
WPT312-11-1	*DCL1a/DCL1a/dcl1b*^Δ^*^15^/dcl1b*^Δ^*^15^*	M_1_	None
WPT312-5-5	*dcl1a*^Δ^*^7^/dcl1a*^Δ^*^7^/DCL1b/dcl1b*^Δ^*^3^*	M_1_	None
WPT312-5-25	*dcl1a*^Δ^*^6^/dcl1a*^Δ^*^6^/DCL1b/dcl1b*^Δ^*^3^*	M_1_	None
WPT312-5-56	*dcl1a*^Δ^*^6^/dcl1a*^Δ^*^7^/DCL1b/dcl1b*^Δ^*^3^*	M_1_	None
WPT312-5-5-1	*dcl1a*^Δ^*^7^/dcl1a*^Δ^*^7^/dcl1b*^Δ^*^3^/dcl1b*^Δ^*^3^*	M_2_	None
M14-533-1	*DCL1a/dcl1a*^Δ^*^7^/DCL1b/dcl1b*^Δ^*^15^*	M_1_F_1_	None
M14-533-2 & 3	*DCL1a/dcl1a*^Δ^*^7^/dcl1b*^Δ^*^3^/dcl1b*^Δ^*^15^*	M_1_F_2_	None
M14-534-1 & 2	*DCL1a/dcl1a*^Δ^*^7^/dcl1b*^Δ^*^3^/dcl1b*^Δ^*^15^*	M_1_F_2_	None
M14-533-1-1	*dcl1a*^Δ^*^7^/dcl1a*^Δ^*^7^/ dcl1b*^Δ^*^15^/dcl1b*^Δ^*^15^*	M_1_F_2_	None
M14-533-2-1	*dcl1a*^Δ^*^7^/dcl1a*^Δ^*^7^/ dcl1b*^Δ^*^3^/dcl1b*^Δ^*^15^*	M_1_F_2_	None

A series of self-pollination and cross-fertilization experiments were conducted to isolate single homozygous mutant individuals and combine *dcl1a* and *dcl1b* alleles in a *dcl1a*/*dcl1b* double mutant (Figure 2, A and B). This approach resulted in the identification of several homozygous single mutants including plants with the allele combinations of *dcl1a*^Δ^*^7^/dcl1a*^Δ^*^7^/DCL1b/DCL1b*, *dcl1a*^Δ^*^6^/dcl1a*^Δ^*^6^/DCL1b/DCL1b*, *DCL1a/DCL1a/dcl1b*^Δ^*^3^/dcl1b*^Δ^*^3^*, and *DCL1a/DCL1a/dcl1b*^Δ^*^15^/dcl1b*^Δ^*^15^*. Furthermore, several double mutant combinations were also identified, including plants with the allele combinations *dcl1a*^Δ^*^7^/dcl1a*^Δ^*^7^/dcl1b*^Δ^*^3^/dcl1b*^Δ^*^15^* and the homozygous genotypes *dcl1a*^Δ^*^7^/dcl1a*^Δ^*^7^/dcl1b*^Δ^*^3^/dcl1b*^Δ^*^3^*, and *dcl1a*^Δ^*^7^/dcl1a*^Δ^*^7^/dcl1b*^Δ^*^15^/dcl1b*^Δ^*^15^* (File S3 and Table 1).

### Phenotypic analysis of single and double dcl1 mutants

**Figure 3:**
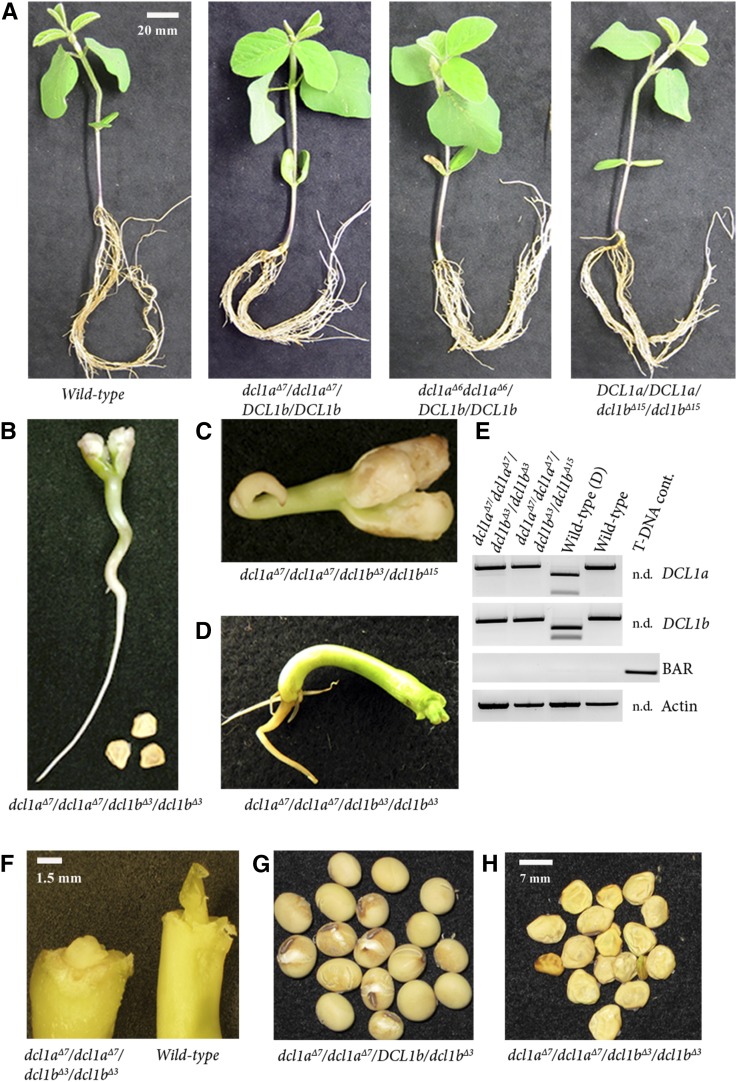
Phenotypic analysis of single and double *dcl1* mutants. (A) Fourteen-day-old single homozygous *dcl1a* or *dcl1b* mutant plants exhibiting no obvious developmental abnormalities. (B–D) Developmental abnormalities were observed in 14-day-old *dcl1a*/*dcl1b* double mutant plants: *dcl1a*^Δ^*^7^/dcl1a*^Δ^*^7^/dcl1b*^Δ^*^3^/ dcl1b*^Δ^*^3^* and *dcl1a*^Δ^*^7^/dcl1a*^Δ^*^7^/dcl1b*^Δ^*^3^/dcl1b*^Δ^*^15^*. A *dcl1a*^Δ^*^7^/dcl1a*^Δ^*^7^/dcl1b*^Δ^*^3^/dcl1b*^Δ^*^3^* plant with removed cotyledons revealed an apical meristem that could not differentiate. Heterozygote plants with a wild-type allele of *DCL1b* had a wild-type appearance. (E) PCR restriction-digestion assays showed that both assessed plants (B–D) are double-mutants (PCR bands are resistant to digestion) and that they are nontransgenic (no band was amplified in the BAR PCR reaction, while a positive control transgenic plant produced the BAR band). (F) Four-day-old *dcl1a*^Δ^*^7^/dcl1a*^Δ^*^7^ /dcl1b*^Δ^*^3^/dcl1b*^Δ^*^3^* mutant and wild-type seedlings after removal of the cotyledons to expose the shoot apical meristem (SAM). The mutant SAM could not initiate leaves, presumably due to defective miRNA processing. (G–H) Seed phenotypes displayed by *dcl1a*^Δ^*^7^/dcl1a*^Δ^*^7^/DCL1b/dcl1b*^Δ^*^3^* and *dcl1a*^Δ^*^7^/dcl1a*^Δ^*^7^/dcl1b*^Δ^*^3^/dcl1b*^Δ^*^3^* plants. Seed from the plant with one wild-type *DCL1b* allele, *dcl1a*^Δ^*^7^/dcl1a*^Δ^*^7^/DCL1b/dcl1b*^Δ^*^3^* genotype, exhibit a wild-type phenotype while seed of the homozygous double-mutant plant, *dcl1a*^Δ^*^7^/dcl1a*^Δ^*^7^/dcl1b*^Δ^*^3^/dcl1b*^Δ^*^3^*, are shrunken and shriveled and can only germinate under specific induction conditions.

Phenotypic analyses were performed on the single homozygous mutant plants *dcl1a*^Δ^*^7^/dcl1a*^Δ^*^7^/DCL1b/DCL1b*, *dcl1a*^Δ^*^6^/dcl1a*^Δ^*^6^/DCL1b/DCL1b*, and *DCL1a/ DCL1a/dcl1b*^Δ^*^15^/dcl1b*^Δ^*^15^*. These plants were evaluated for developmental abnormalities, including height differences, and/or altered nodulation characteristics. No noticeable phenotype was observed in any of the analyzed single mutants (Figure 3A), revealing no evidence for subfunctionalization or neofunctionalization of the soybean *DCL1* homologs.

The previous segregation analysis of the progeny of T_0_ plant WPT312-5 (*dcl1a*^Δ^*^6^/dcl1a*^Δ^*^7^/DCL1b/dcl1b*^Δ^*^3^*) did not identify a viable homozygous double mutant *dcl1* plant (Figure 2A). Approximately one-sixteenth of segregating T_1_ seed failed to germinate with the planted seeds consequently rotting in soil. This strongly indicated that plants harboring the double *dcl1* mutation had an embryonic lethal phenotype, or that the mutations exacerbated embryonic dormancy. To rule out seed dormancy, we introduced a seed stratification step, namely 5 d incubation at 4° on sterile filter paper, followed by 3 d room temperature incubation in the dark. Two d poststratification, three seeds germinated, as evidenced by the emergence of the embryonic axis. The seed coats were gently removed to facilitate complete germination.

The stratification, incubation, and seed coat removal steps allowed for the observation of early developmental phenotypes in *dcl1* double mutants. Two different double mutant genotypes were evaluated, including the *dcl1a*^Δ^*^7^/dcl1a*^Δ^*^7^/dcl1b*^Δ^*^3^/dcl1b*^Δ^*^3^* and *dcl1a*^Δ^*^7^/dcl1a*^Δ^*^7^/dcl1b*^Δ^*^3^/dcl1b*^Δ^*^15^* backgrounds. Seedlings grew to approximately 5–7 cm in height and exhibited striking developmental phenotypes. These included a lopsided differentiation of the vascular tissue, a hallmark of miRNA deregulation (Lobbes *et al.* 2006; Allen *et al.* 2007; Eamens *et al.* 2012), resulting in a wavy stem phenotype. There was also no sign of radial root growth or leaf initiation at the shoot apical meristem. Removal of the atrophied cotyledons revealed a shoot apical meristem that was either delayed or not capable of shoot initiation (Figure 3, B–D). A PCR digestion assay revealed digestion-resistant amplicons from the *DCL1a* and *DCL1b* loci, confirming the biallelic mutations harbored by the plant (Figure 3E). Sequencing of these amplicons confirmed the genotype status of the double mutation (File S3). In addition, apical meristem disruption and a shrunken seed phenotype were readily observed to correlate with double-mutant genotypes (Figure 3, F–H).

### Assessment of miRNA processing and function in single and double dcl1 mutants

**Figure 4:**
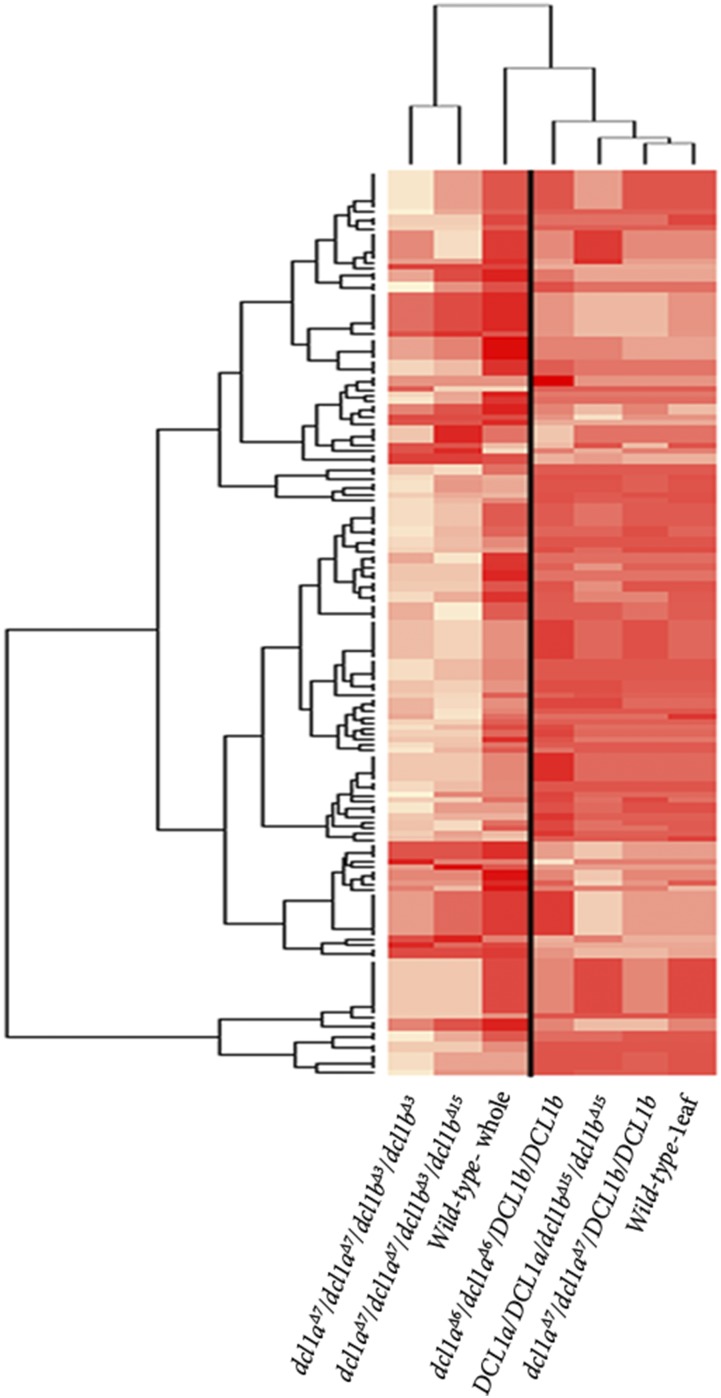
Small RNA sequence profiling of miRNA accumulation in single and double *dcl1* mutants. Small RNA sequence profiling of soybean miRNAs in 14-day-old single and double *dcl1* mutant plants. The rows indicate miRNA accumulation in each mutant plant (Table S1 and Table S2).

miRNAs require *DCL1* for their maturation from their hairpin structured precursor transcripts. Upon maturation, miRNAs are key regulators of gene expression in development and in responses to the environment, such as abiotic stress or pathogen attack (Liu *et al.* 2008). Furthermore, disrupted miRNA biogenesis is a hallmark of previously characterized *dcl1* mutants in multiple plant species (Schauer *et al.* 2002; Liu *et al.* 2005; Valdes-Lopez *et al.* 2008; Khraiwesh *et al.* 2010; Thompson *et al.* 2014). We therefore expected to detect a global reduction of total miRNA accumulation, combined with globally elevated miRNA target gene expression in our soybean *dcl1* mutants. To test this, sRNA populations were profiled from two biological replicates of 14-day-old leaf material for wild-type plants and the homozygous single mutants; *dcl1a*^Δ^*^7^/dcl1a*^Δ^*^7^/DCL1b/DCL1b*, *dcl1a*^Δ^*^6^/dcl1a*^Δ^*^6^/DCL1b/DCL1b*, and *DCL1a/ DCL1a/dcl1b*^Δ^*^15^/dcl1b*^Δ^*^15^*. The mutants exhibited minimal differences in miRNA levels compared with the wild-type control, indicating probable functional redundancy between the homologous *DCL1a* and *DCL1b* genes (Figure 4 and Table S1).

A similar comparison was performed on the double mutant backgrounds *dcl1a*^Δ^*^7^/dcl1a*^Δ^*^7^/dcl1b*^Δ^*^3^/dcl1b*^Δ^*^3^* and *dcl1a*^Δ^*^7^/dcl1a*^Δ^*^7^/dcl1b*^Δ^*^3^/dcl1b*^Δ^*^15^*. However, since the double mutants only produced cotyledons, stems, and roots, the sRNA fraction was instead extracted from available 14-day-old ‘whole plant’ materials, for both the wild-type and mutants. Analysis of the double mutants revealed an almost global reduction in miRNA accumulation, ranging from negligible to a maximum 7.5-fold difference, indicating a vast reduction in mature miRNA levels in the double mutants (Figure 4 and Table S2). Among the most highly reduced miRNAs were the closely related miRNAs miR319f and miR159a, miRNAs that target a subset of genes encoding the developmentally important TCP and GAMYB-like transcription factors, respectively.

**Figure 5:**
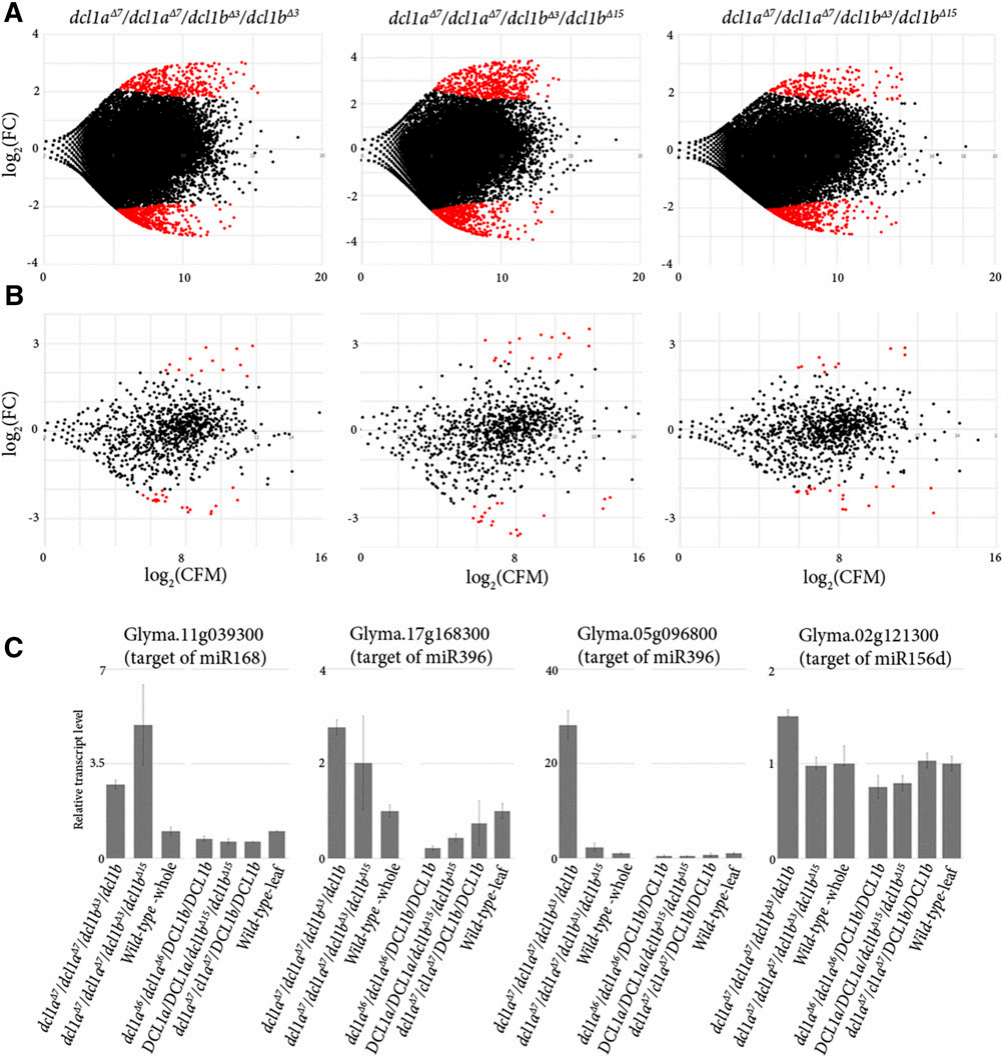
The analysis of predicted miRNA targets in the double *dcl1* mutant. (A) Three MA plots of the *dcl1* double mutants with one replicate of mutant *dcl1a*^Δ^*^7^/dcl1a*^Δ^*^7^/dcl1b*^Δ^*^3^/dcl1b*^Δ^*^3^* and two replicates of mutant *dcl1a*^Δ^*^7^/dcl1a*^Δ^*^7^/dcl1b*^Δ^*^3^/dcl1b*^Δ^*^15^*. The plots show the differentially expressed transcripts in the *dcl* double mutant compared to wild-type, with the red dots indicating significantly perturbed transcript expression (*P* <0.05). (B) Three MA plots indicating differential expression for a subset of transcripts in (A). This subset includes only genes validated as miRNA target transcripts. A total of 985, 977 and 981 distinct miRNA targets were found to be expressed in each of the three assessed mutants, respectively. The red dots indicate the 35, 38 and 30 differentially expressed miRNA target transcripts (*P* <0.05) in each mutant when compared to wild-type controls. (C) RT-qPCR was used to determine the expression of a subset of miRNA target transcripts in single and double *dcl1* mutant backgrounds. CPM, counts per million; FC, fold change.

We next performed standard RNA-Seq (mRNA profiling) on the same *dcl1* double mutant samples in order to identify and quantify differential expression of target transcripts in the double mutants. This analysis identified approximately 844 transcripts with significantly altered expression (*P* <0.05) from 34,959 transcripts detected in the *dcl1a*^Δ^*^7^/dcl1a*^Δ^*^7^/dcl1b*^Δ^*^3^/dcl1b*^Δ^*^3^* mutant, 1034 transcripts from the 34,830 transcripts detected for the *dcl1a*^Δ^*^7^/dcl1a*^Δ^*^7^/dcl1b*^Δ^*^3^/dcl1b*^Δ^*^15^* mutant, and 735 significantly (*P* <0.05) altered transcripts from the 34,965 transcripts detected from the second *dcl1a*^Δ^*^7^/dcl1a*^Δ^*^7^/dcl1b*^Δ^*^3^/dcl1b*^Δ^*^15^* mutant (Figure 5A and Table S3). To improve the resolution of these plots, we carried out an analysis that identified predicted miRNA target transcripts using the *sPARTA* package (Kakrana *et al.* 2014) and combined publicly available PARE (Parallel Analysis of RNA Ends) libraries (Arikit *et al.* 2014) to map these miRNA target transcripts to the differentially expressed transcripts depicted in Figure 5A. This approach identified a total of 985, 977, and 981 distinct miRNA target transcripts in the RNA-Seq datasets analyzed from the three assessed mutant backgrounds and approximately 35, 38, and 30 of these were determined significantly differentially expressed (*P* <0.05), respectively. Considerable overlap was observed for the differentially expressed miRNA target genes between the different mutant backgrounds assessed (Figure 5B, Table S4, and Table S5). Finally, quantitative reverse transcription PCR (RT-qPCR) was used to confirm the expression of a small subset of previously published miRNA target transcripts (Kulcheski *et al.* 2011) and this quantitative analysis aligned closely with the RNA-Seq generated data. Intriguingly, some miRNA target transcripts in the *dcl1a*^Δ^*^7^/dcl1a*^Δ^*^7^/dcl1b*^Δ^*^3^/dcl1b*^Δ^*^3^* mutant background, notably the miR396 target gene Glyma05g20930 (Glyma.05g096800 in the version 2 genome assembly), remained at wild-type levels. This curious finding suggests that the *dcl1b*^Δ^*^15^* mutant allele might function differently to the *dcl1b*^Δ^*^3^* allele on specific miRNA-associated substrates.

## Discussion

Functional genomic studies in soybean can be challenging due to the duplicated paleopolyploid nature of the soybean genome. Gene redundancy among homologous copies is often sufficient for full complementation of the loss of one homolog. The soybean *DCL1* homologs are almost identical to one another at the nucleotide level and, further, have almost identical expression patterns in most soybean tissues. Our data shows that miRNA biogenesis is largely unaffected in plant lines harboring a mutation in one of the two soybean *DCL1* loci. This strongly suggests that the *DCL1a* and *DCL1b* homologs perform redundant functions in the soybean miRNA pathway. This is in direct contrast to a recent report in *P. patens* (Khraiwesh *et al.* 2010) where the two highly similar DCL1 enzymes were shown to perform highly specialized roles at functionally distinct stages of the *P. patens* miRNA pathway. This strongly suggests that neofunctionalization of an ancient *P. patens* DCL1 occurred prior to the duplication of this ancestral gene (Khraiwesh *et al.* 2010).

The isolation of a *dcl1* double mutant was required to overcome the hurdle of *DCL1a/DCL1b* redundancy, and to confirm a role for each DCL1 homolog in the soybean miRNA pathway. To achieve this goal, ZFN-based mutagenesis allowed for the production and recovery of stable, nontransgenic *dcl1a* and *dcl1b* mutants, and the subsequent generation of multiple allelic combinations of the *dcl1a/dcl1b* double mutant. The first double mutant identified in this study was isolated from a single T_0_ plant that harbored mutations targeted to three of the four *DCL1* loci. This included distinct mutations for each of the *DCL1a* homologs (*i.e.*, biallelic) and a monoallelic mutation for *DCL1b*. The second double mutant allele was generated by traditional cross-fertilization of the *DCL1a/DCL1a/dcl1b*^Δ^*^15^/dcl1b*^Δ^*^15^* mutant with the *dcl1a*^Δ^*^7^/dcl1a*^Δ^*^7^/DCL1b/dcl1b*^Δ^*^3^* mutant.

While frameshift *dcl1a* mutant alleles were identified in this work, there have been no out-of-frame *dcl1b* mutant alleles identified to date. Both *dcl1b* alleles from this study were in-frame mutations that result from a one and a five amino acid residue deletion, respectively. Therefore, at this stage, we cannot confirm whether an out-of-frame single *dcl1b* mutant alone would be sufficient to disrupt the soybean miRNA pathway. However, the in-frame *dcl1b* mutations clearly disrupt protein function, as the phenotypically normal *dcl1a*^Δ^*^7^/dcl1a*^Δ^*^7^/DCL1b/DCL1b* single mutant exhibits severe seedling phenotypes when combined with the *dcl1b* in-frame mutations. Furthermore, initial screening of the *dcl1a*^Δ^*^6^/dcl1a*^Δ^*^7^/DCL1b/dcl1b*^Δ^*^3^* T_0_ plant failed to recover viable double mutants with combinations of the in-frame mutant alleles (*dcl1a*^Δ^*^6^* and *dcl1b*^Δ^*^3^*). This would suggest that in-frame mutations also appear to disrupt the function of DCL1, otherwise at least one or more viable *dcl1a*^Δ^*^6^/dcl1a*^Δ^*^6^/dcl1b*^Δ^*^3^/dcl1b*^Δ^*^3^* or heterozygote variations of the double mutant would have been recovered. An out-of-frame *dcl1b* mutation combined with the *dcl1a*^Δ^*^7^* allele would likely be embryo lethal and not viable. Such a plant would be consistent with the previously reported embryo defective *Arabidopsis dcl1-4* (*sus1-4*) mutant resulting from a T-DNA insertion disrupting the PAZ domain (McElver *et al.* 2001).

We also performed small RNA sequencing to establish miRNA accumulation profiles for the three single *dcl1* mutant alleles, including mutant lines *dcl1a*^Δ^*^7^/dcl1a*^Δ^*^7^/DCL1b/DCL1b* (out-of-frame mutation), *dcl1a*^Δ^*^6^/dcl1a*^Δ^*^6^/DCL1b/ DCL1b* (in-frame mutation), and *DCL1a/ DCL1a/dcl1b*^Δ^*^15^/dcl1b*^Δ^*^15^* (in-frame mutation). Compared to wild-type plants, no major alterations to miRNA accumulation were observed in any of the analyzed single mutants. However, in the three assessed double mutants, we observed an almost global reduction in miRNA accumulation. Intriguingly, miRNA accumulation was not uniformly reduced, or completely abolished in any assessed double mutant, including several miRNAs that are largely unaffected in these backgrounds. Possible explanations for this curious finding include: 1) disruption of the so-called Dicer ’molecular ruler’; 2) disassociation of various interacting proteins that either bind to single and double-stranded transcripts or to DCL1 itself; 3) gene redundancies between the five other soybean *DCL* paralogs (Curtin *et al.* 2012); or 4) a defective DCL1b mutant protein that is still functionally competent to process a small subset of miRNAs (MacRae *et al.* 2006; Rogers and Chen 2013). We discuss each of these possibilities in more detail below.

Previous crystal structure analysis of the Dcr enzyme revealed a region between the PAZ and the first RNase III domain that essentially functions as a ‘molecular ruler’ by governing the size of the processed sRNAs (MacRae *et al.* 2006). The *dcl1b* mutation could have potentially disrupted this ruler resulting in shorter, misprocessed miRNAs. However, the small RNA sequencing analysis was able to distinguish sRNA sizes ranging from 18 –35 nt and there was no indication of short miRNA sequences in either the single or double mutant datasets. This suggests that disruption to the molecular ruler of the two soybean DCL1s is unlikely to have been the cause of incomplete loss of miRNA accumulation. The precise molecular mechanisms of how DCL1 recognizes, interacts with, and processes precursor molecules, namely primary-miRNAs (pri-miRNAs) and precursor-miRNAs (pre-miRNAs) to miRNA/miRNA* duplexes is not completely understood. Two protein cofactors, including the double-stranded (ds)RNA-binding protein DRB1 (HYL1) and the C2H2-type zinc finger domain-containing protein SERRATE (SE), have been shown to interact with both DCL1 and pri-miRNAs for accurate and efficient processing of miRNAs from their precursor transcripts (Rogers and Chen 2013). It is plausible that interactions between DCL1 and these protein cofactors are disrupted by the *dcl1b* mutations. In turn, this could affect the loading of the duplex miRNA into the RISC by SE or DRB1, or the accuracy of DCL1-mediated pri-miRNA and pre-miRNA processing (Eamens *et al.* 2009; Rogers and Chen 2013).

Another possible explanation for incomplete loss of miRNA accumulation in the double mutant is gene redundancy by other DCL enzymes such as DCL2 or DCL4. There are seven functional DCL enzymes in soybean including two DCL2 (DCL2a and DCL2b) and two DCL4 (DCL4a and DCL4b) proteins (Curtin *et al.* 2012). Reports from *Arabidopsis* have shown that the DCL4/DRB4 functional partnership can process certain miRNAs (Rajagopalan *et al.* 2006). This possibility is further supported by other demonstrations in *Arabidopsis*; that is, DRB2 appears capable of forming functional partnerships with either DCL1 or DCL4 for miRNA and siRNA production, respectively (Eamens *et al.* 2012). Therefore, the higher than expected levels observed for some miRNAs in the double mutant backgrounds may result from DCL4 activity, with the functional assistance of either DRB2 or DRB4, partnerships that would have increased access to miRNA precursor transcripts in the absence of functionally competent DCL1a and DCL1b.

An additional explanation involves irregularities with the plant pri-miRNA processing step. Typically, pri-miRNAs are cleaved by the DCL1 enzyme in the canonical ‘base-to-loop’ manner; the first cleavage event is at the base of the hairpin to produce the pre-miRNA, and the second cleavage event liberates the miRNA/miRNA* duplex from the loop structure of the pre-miRNA hairpin. In plants, there is also a highly conserved, yet ‘noncanonical’ pathway that is initiated and proceeds in the opposite direction to the two-step canonical pri-miRNA processing pathway. In the noncanonical ‘loop-to-base’ pri-miRNA processing pathway, four DCL1-mediated cleavage steps are required for pri-miRNA to pre-miRNA processing, and subsequent miRNA/miRNA* duplex liberation of the pre-miRNA. Currently, only two *Arabidopsis* miRNAs have been demonstrated to be processed by this more complex pathway, including miR159 and mR319 (Bologna *et al.* 2009). Coincidentally, the two most differentially expressed miRNAs in our data set are miR159 and miR319, a finding that suggests that the noncanonical pri-miRNA processing pathway may be more severely affected in the *dcl1a*/*dcl1b* double mutant background. If this is the case, then other miRNAs in our data set with highly differentiated expression states, such as miR403a, miR403b, miR1535b, may also be processed by the noncanonical loop-to-base pathway.

Lastly, RNA-Seq of the double mutant and wild-type plants provides insight into the extent of miRNA accumulation and target gene disruption in these plants. Altered expression of protein-encoding genes that would normally be targets of miRNA regulation was observed in each of the three assessed double mutants, including 35, 38, and 30 putative miRNA target genes, respectively. However, there were approximately 980 putative miRNA protein-encoding target genes identified in total in these analyses. Furthermore, considerable overlap of these differentially expressed miRNA target genes was observed across the assessed mutant backgrounds. Therefore, these results indicate that ∼3%–5% of the total predicted gene targets for each of the currently known soybean miRNAs were differentially expressed. Several possible explanations might account for this low number of affected target transcripts. These include insufficient sample replication resulting in diminished statistical power for the differential expression analysis, thereby contributing to an increase occurrence of false negatives. In addition, it is possible (though unlikely given the severity of the mutant phenotype) that a hypomorphic DCL1 enzyme remains active on some substrates. However, the severe developmental phenotype expressed by the double mutants (*i.e.*, no leaves were available for sampling, with only stem, root, and shriveled cotyledons available for double mutant analyses) makes the differential expression analysis difficult to fully interpret. A future experiment focused on a well-replicated and microdissected SAM, or on germinating seed tissue, would likely yield an improved and more biologically relevant indication of the differentially expressed miRNA target transcript landscape resulting from the introduced mutations.

In conclusion, this study demonstrates targeted mutagenesis using site-specific nucleases as a reverse genetics tool for functional genomics studies in soybean. We generated *dcl1a/dcl1b* double mutants and demonstrated that the introduction of mutations into both *DCL1* loci severely disrupted the soybean miRNA pathway, including altered miRNA accumulation and deregulated miRNA target gene expression. The work presented here provides the soybean community with an important genetic resource to aid in the further study of miRNA-directed RNA silencing-mediated processes, including mounting defense responses against abiotic stress and plant viral, fungal, oomycete, insect, and bacterial pathogens. For example, soybean plants grown in the Mid-West of the United States are highly susceptible to the stem and root rot pathogen *Phytopthora sojae*, causing millions of dollars in lost revenue annually. It was recently demonstrated that *P. sojae* secretes proteins that suppress RNA silencing pathways of the host plant, namely the miRNA pathway, to aid in the pathogen’s infection and colonization of soybean (Qiao *et al.* 2013). The current mechanism for how *P. sojae* subverts the RNA silencing pathways in soybean is unknown, but genetic resources such as *dcl1* and *dcl4* mutants may be important for the elucidation of this plant-pathogen interaction. Furthermore, mutant alleles for the remaining soybean *DCL* genes may be generated using more efficient SSN platforms in the future. This will be critical for generating double mutants in what would otherwise be a difficult and time-consuming undertaking using the more traditional mutagenesis, mutation identification, and stacking methodologies. The ZFN and TALEN SSN platforms are fast becoming outdated due to the ease, efficiency and multiplex capabilities of the CRISPR/Cas9 platform, which will likely be the ‘go-to’ platform in the foreseeable future (Baltes and Voytas 2015).

## Supplementary Material

Supporting Information
