# Supplementary material for: MicroRNA Maturation and MicroRNA Target Gene Expression Regulation Are Severely Disrupted in Soybean dicer-like1 Double Mutants
Source: G3 (Bethesda). 2015 Dec 15;6(2):423–33. doi: 10.1534/g3.115.022137 (PMC4751560; doi:10.1534/g3.115.022137)
Supplement: Supporting Information [file supp_6_2_423__index.html]

MicroRNA Maturation and MicroRNA Target Gene Expression Regulation Are Severely Disrupted in Soybean dicer-like1 Double Mutants — Supporting Information 

# MicroRNA Maturation and MicroRNA Target Gene Expression Regulation Are Severely Disrupted in Soybean *dicer-like1* Double Mutants

## Supporting Information for Curtin *et al.*, 2016

**Files in this Data Supplement:**

- File S1 - (A) The DCL1 zinc-finger nuclease target site and the individual seven amino acid zinc-finger cassettes (F1, F2 & F3) that make-up each zinc-finger array (Sander *et al.*, 2011). (.pdf, 379 KB)
- File S2 - (A) Sequence confirmation of single *dcl1a* and *dcl1b* mutants WPT312-5-11 *dcl1aΔ7/dcl1aΔ7/DCL1b/DCL1b*. (.pdf, 345 KB)
- File S3 - (A) Sequence confirmation of the double mutant *dcl1aΔ7/dcl1aΔ7/ dcl1bΔ3/dcl1bΔ3*. (.pdf, 462 KB)
- Table S1 - miRNA abundances in the single *dcl1a* and *dcl1b* mutant libraries. (.xlsx, 64 KB)
- Table S2 - miRNA abundances in the double *dcl1a/dcl1b* mutant libraries. (.xlsx, 48 KB)
- Table S3 - mRNA-seq reads of total transcripts in the double *dcl1a/dcl1b* mutant libraries. (.xlsx, 23,125 KB)
- Table S4 - mRNA-seq reads of predicted miRNA target transcripts in the double *dcl1a/dcl1b* mutant libraries. (.xlsx, 12,265 KB)
- Table S5 - Soybean miRNAs and their predicted target transcripts. (.csv, 3,133 KB)
