# Supplementary material for: MicroRNA Maturation and MicroRNA Target Gene Expression Regulation Are Severely Disrupted in Soybean dicer-like1 Double Mutants
Source: G3 (Bethesda). 2015 Dec 15;6(2):423–33. doi: 10.1534/g3.115.022137 (PMC4751560; doi:10.1534/g3.115.022137)
Supplement: Supporting Information [file supp_g3.115.022137_FileS1.pdf]

A

GOI	Promoter	Architecture	Accession	Target site	ZFAs
DCL1a	Inducible	ZFN-4R	Glyma03g42290	cAGCAACCTCTTATAAGAGGGCGTGg	TKQILGR HKSSLTR RHDQLTR
DCL1b	Inducible	ZFN-4R	Glyma19g45060	gTCGTTGGAGAATATTCTCCCGCACc	SRFTLGR LKEHLTR RVDNLPR

B

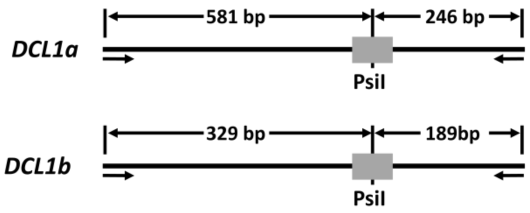

C

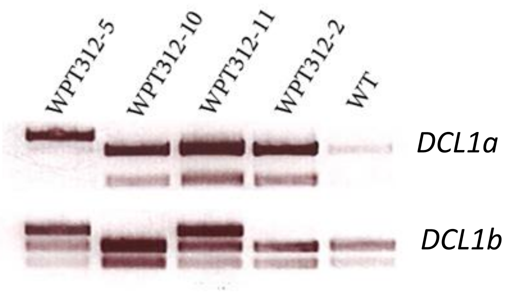

D

WPT312-5_T0 _*DCL1a*

```
GGGGTTAATTTGATTTACAAGCAGCAA-------TAAGAGGGCGTGGTGTATCATACTGCAAGAATCT  Δ7-bp
GGGGTTAATTTGATTTACAAGCAGCAA-------TAAGAGGGCGTGGTGTATCATACTGCAAGAATCT  Δ7-bp
GGGGTTAATTTGATTTACAAGCAGCAACCTCTT------GGGCGTGGTGTATCATACTGCAAGAATCT  Δ6-bp
GGGGTTAATTTGATTTACAAGCAGCAACCTCTT------GGGCGTGGTGTATCATACTGCAAGAATCT  Δ6-bp
GGGGTTAATTTGATTTACAAGCAGCAA-------TAAGAGGGCGTGGTGTATCATACTGCAAGAATCT  Δ7-bp
GGGGTTAATTTGATTTACAAGCAGCAACCTCTT------GGGCGTGGTGTATCATACTGCAAGAATCT  Δ6-bp
GGGGTTAATTTGATTTACAAGCAGCAACCTCTTATAAGAGGGCGTGGTGTATCATACTGCAAGAATCT  WT
```

WPT312-5 _T0_*DCL1b*

```
GGGGTTGATTTGATTTACAGGCAGCAACCTCT---AAGAGGGCGTGGTGTATCATACTGCAAGAATCT  Δ3-bp
GGGGTTGATTTGATTTACAGGCAGCAACCTCT---AAGAGGGCGTGGTGTATCATACTGCAAGAATCT  Δ3-bp
GGGGTTGATTTGATTTACAGGCAGCAACCTCT---AAGAGGGCGTGGTGTATCATACTGCAAGAATCT  Δ3-bp
GGGGTTGATTTGATTTACAGGCAGCAACCTCTTATAAGAGGGCGTGGTGTATCATACTGCAAGAATCT  WT
```

WPT312-11 _T0_*DCL1b*

```
GGGGTTGATTTGATTTACAGGCAGCAACCTCT---------------TGTATCATACTGCAAGAATCT  Δ15-bp
GGGGTTGATTTGATTTACAGGCAGCAACCTCT---------------TGTATCATACTGCAAGAATCT  Δ15-bp
GGGGTTGATTTGATTTACAGGCAGCAACCTCT---------------TGTATCATACTGCAAGAATCT  Δ15-bp
GGGGTTGATTTGATTTACAGGCAGCAACCTCTTATAAGAGGGCGTGGTGTATCATACTGCAAGAATCT  WT
```

File S1. (A) The DCL1 zinc-finger nuclease target site and the individual seven amino acid zinc-finger cassettes (F1,F2 & F3) that make-up each zinc-finger array [23].**(B)** PCR assays for detection of mutated *DCL1a* and *DCL1b* alleles. PsiI resistant amplicons indicate target mutations. **(C)** PCR of WPT312-5, WPT312-10, WPT312-11 and WPT312-2 T0 plants.. The gel bands indicate bi-allelic mutations at *DCL1a* and a mono-allelic mutation at *DCL1b* in the WPT312-5 plant. A mono-allelic mutation of *DCL1b* was observed in WPT312-11. Both WPT312-10, WPT312-2 plants had wild-type alleles at both *DCL1a* and *DCL1b* loci. **(D)** Sequence confirmation of *dcl1a* and *dcl1b* mutant alleles.
