# Supplementary material for: MicroRNA Maturation and MicroRNA Target Gene Expression Regulation Are Severely Disrupted in Soybean dicer-like1 Double Mutants
Source: G3 (Bethesda). 2015 Dec 15;6(2):423–33. doi: 10.1534/g3.115.022137 (PMC4751560; doi:10.1534/g3.115.022137)
Supplement: Supporting Information [file supp_g3.115.022137_FileS2.pdf]

A

WPT312-5-11 _*DCL1a*_Δ7-bp_PCR un-enriched template

```
GGGGTTAATTTGATTTACAAGCAGCAA-------TAAGAGGGCGTGGTGTATCATACTGCAAGAATCT  Δ7-bp
GGGGTTAATTTGATTTACAAGCAGCAA-------TAAGAGGGCGTGGTGTATCATACTGCAAGAATCT  Δ7-bp
GGGGTTAATTTGATTTACAAGCAGCAA-------TAAGAGGGCGTGGTGTATCATACTGCAAGAATCT  Δ7-bp
GGGGTTAATTTGATTTACAAGCAGCAA-------TAAGAGGGCGTGGTGTATCATACTGCAAGAATCT  Δ7-bp
GGGGTTAATTTGATTTACAAGCAGCAACCTCTTATAAGAGGGCGTGGTGTATCATACTGCAAGAATCT  WT
```

WPT312-5-11 _ *DCL1b*_no deletion_PCR.*Psi*I amplicon

```
GGGGGTGGATTGGATTTCCAGGCAGCAACTTCTTATAAGAGGGCGTGGTGTATCATACTGCAAGAATCTT  WT
GGGGGTGGATTTGATTTCCAGGCAGCAACCTCTTATAAGAGGGCGTGGTGTATCATACTGCAAGAATCTT  WT
GGGGGTGGATTTGATTTCCAGGCAGCAACTTCTTATAAGAGGGCGTGGTGTATCATACTGCAAGAATCTT  WT
GGGGGTGGATTGGATTTCCAGGCAGCAACTTCTTATAAGAGGGCGTGGTGTATCATACTGCAAGAATCTT  WT
TGGGGTTGATTTGATTTACAGGCAGCAACCTCTTATAAGAGGGCGTGGTGTATCATACTGCAAGAATCTT  WT
```

Status*: (dcl1a* $^{\Delta7}$*dc1a* $^{\Delta7}$*/DCL1b/DCL1b*) without transgene

B

WPT312-5-36_*DCL1a*_Δ6-bp_PCR un-enriched template

```
GGGGTTAATTTGATTTACAAGCAGCAACCTCTT------GGGCGTGGTGTATCATACTGCAAGAATCT  Δ6-bp
GGGGTTAATTTGATTTACAAGCAGCAACCTCTT------GGGCGTGGTGTATCATACTGCAAGAATCT  Δ6-bp
GGGGTTAATTTGATTTACAAGCAGCAACCTCTT------GGGCGTGGTGTATCATACTGCAAGAATCT  Δ6-bp
GGGGTTAATTTGATTTACAAGCAGCAACCTCTT------GGGCGTGGTGTATCATACTGCAAGAATCT  Δ6bp
GGGGTTAATTTGATTTACAAGCAGCAACCTCTTATAAGAGGGCGTGGTGTATCATACTGCAAGAATCT  WT
```

WPT312-5-36_*DCL1b*_ no deletion_PCR.*Psi*I amplicon

```
GGGGTGGATTTGATTTACAGGCAGCAACCTCTTATAAGAGGGCGTGGTGTATCATACTGCAAGAATCT  WT
GGGGTGGATTTGATTTACAGGCAGCAACCTCTTATAAGAGGGCGTGGTGTATCATACTGCAAGAATCT  WT
GGGGTGGATTGGATTTACAGGCAGCAACCTCTTATAAGAGGGCGTGGTGTATCATACTGCAAGAATCT  WT
GGGGTGGATTTGATTAACAGGCAGCAACCTCTTATAAGAGGGCGTGGTGTATCATACTGCAAGAATCT  WT
GGGGTTGATTTGATTTACAGGCAGCAACCTCTTATAAGAGGGCGTGGTGTATCATACTGCAAGAATCT  WT
```

Status*: (dcl1a* $^{\Delta6}$*dc1a*$^{\Delta6}$*/DCL1b/DCL1b*) without transgene

C

WPT312-11-1_*DCL1a*_no deletion_PCR.*Psi*I amplicon

```
GGGGTTAATTTGATTTACAAGCAGCAACCTCTTATAAGAGGGCGTGGTGTATCATACTGCAAGAATCT  WT
GGGGTTAATTTGATTTACAAGCAGCAACCTCTTATAAGAGGGCGTGGTGTATCATACTGCAAGAATCT  WT
GGGGTTAATTTGATTTACAAGCAGCAACCTCTTATAAGAGGGCGTGGTGTATCATACTGCAAGAATCT  WT
GGGGTTAATTTGATTTACAAGCAGCAACCTCTTATAAGAGGGCGTGGTGTATCATACTGCAAGAATCT  WT
GGGGTTAATTTGATTTACAAGCAGCAACCTCTTATAAGAGGGCGTGGTGTATCATACTGCAAGAATCT  WT
```

WPT312-11-1_*DCL1b*_Δ15-bp_PCR un-enriched template

```
GGGGTTGATTTGATTTACAGGCAGCAACCTCT---------------TGTATCATACTGCAAGAATCT  Δ15-bp
GGGGTTGATTTGATTTACAGGCAGCAACCTCT---------------TGTATCATACTGCAAGAATCT  Δ15-bp
GGGGTTGATTTGATTTACAGGCAGCAACCTCT---------------TGTATCATACTGCAAGAATCT  Δ15-bp
GGGGTTGATTTGATTTACAGGCAGCAACCTCT---------------TGTATCATACTGCAAGAATCT  Δ15-bp
GGGGTTGATTTGATTTACAGGCAGCAACCTCTTATAAGAGGGCGTGGTGTATCATACTGCAAGAATCT  WT
```

Status*: (DCL1a/DCL1a/dcl1b*$^{\Delta15}$*/dcl1b*$^{\Delta15}$) without transgene

D

WPT312-5-5 _*DCL1a*_Δ7-bp_PCR un-enriched template

```
GGGGTTAATTTGATTTACAAGCAGCAA-------TAAGAGGGCGTGGTGTATCATACTGCAAGAATCT  Δ7-bp
GGGGTTAATTTGATTTACAAGCAGCAA-------TAAGAGGGCGTGGTGTATCATACTGCAAGAATCT  Δ7-bp
GGGGTTAATTTGATTTACAAGCAGCAA-------TAAGAGGGCGTGGTGTATCATACTGCAAGAATCT  Δ7-bp
GGGGTTAATTTGATTTACAAGCAGCAA-------TAAGAGGGCGTGGTGTATCATACTGCAAGAATCT  Δ7-bp
GGGGTTAATTTGATTTACAAGCAGCAACCTCTTATAAGAGGGCGTGGTGTATCATACTGCAAGAATCT  WT
```

WPT312-5-5 _*DCL1b*_Δ3-bp_PCR.*Psi*I amplicon

```
GGGGTTGATTTGATTTACAGGCAGCAACCTCT---AAGAGGGCGTGGTGTATCATACTGCAAGAATCT  Δ3-bp
GGGGTTGATTTGATTTACAGGCAGCAACCTCTTATAAGAGGGCGTGGTGTATCATACTGCAAGAATCT  WT
GGGGTTGATTTGATTTACAGGCAGCAACCTCT---AAGAGGGCGTGGTGTATCATACTGCAAGAATCT  Δ3-bp
GGGGTTGATTTGATTTACAGGCAGCAACCTCT---AAGAGGGCGTGGTGTATCATACTGCAAGAATCT  Δ3-bp
GGGGTTGATTTGATTTACAGGCAGCAACCTCTTATAAGAGGGCGTGGTGTATCATACTGCAAGAATCT  WT
```

Status: (*dcl1a* $^{\Delta 7}$*dc1a* $^{\Delta 7}$*/DCL1b/dcl1b*$^{\Delta 3}$) without transgene

E

WPT312-5-25_*DCL1a*_Δ6-bp_PCR un-enriched template (see also WPT312-5-121)

```
GGGGTTAATTTGATTTACAAGCAGCAACCTCTT------GGGCGTGGTGTATCATACTGCAAGAATCT  Δ6-bp
GGGGTTAATTTGATTTACAAGCAGCAACCTCTT------GGGCGTGGTGTATCATACTGCAAGAATCT  Δ6-bp
GGGGTTAATTTGATTTACAAGCAGCAACCTCTT------GGGCGTGGTGTATCATACTGCAAGAATCT  Δ6-bp
GGGGTTAATTTGATTTACAAGCAGCAACCTCTT------GGGCGTGGTGTATCATACTGCAAGAATCT  Δ6-bp
GGGGTTAATTTGATTTACAAGCAGCAACCTCTTATAAGAGGGCGTGGTGTATCATACTGCAAGAATCT  WT
```

WPT312-5-25 _Dcl1b_Δ3bp_PCR.*Psi*I amplicon

```
GGGGTTGATTTGATTTACAGGCAGCAACCTCT---AAGAGGGCGTGGTGTATCATACTGCAAGAATCT  Δ3-bp
GGGGTTGATTTGATTTACAGGCAGCAACCTCTTATAAGAGGGCGTGGTGTATCATACTGCAAGAATCT  WT
GGGGTTGATTTGATTTACAGGCAGCAACCTCT---AAGAGGGCGTGGTGTATCATACTGCAAGAATCT  Δ3-bp
GGGGTTGATTTGATTTACAGGCAGCAACCTCTTATAAGAGGGCGTGGTGTATCATACTGCAAGAATCT  WT
GGGGTTGATTTGATTTACAGGCAGCAACCTCTTATAAGAGGGCGTGGTGTATCATACTGCAAGAATCT  WT
```

Status: (*dcl1a* $^{\Delta 6}$*dc1a* $^{\Delta 6}$*/DCL1b/dcl1b*$^{\Delta 3}$) without transgene

F

WPT312-5-56 _*DCL1a*_Δ6-bp and Δ7-bp _PCR un-enriched template (see also WPT312-5-70

```
GGGGTTAATTTGATTTACAAGCAGCAA-------TAAGAGGGCGTGGTGTATCATACTGCAAGAATCT  Δ7-bp
GGGGTTAATTTGATTTACAAGCAGCAACCTCTT------GGGCGTGGTGTATCATACTGCAAGAATCT  Δ6-bp
GGGGTTAATTTGATTTACAAGCAGCAACCTCTT------GGGCGTGGTGTATCATACTGCAAGAATCT  Δ6-bp
GGGGTTAATTTGATTTACAAGCAGCAA-------TAAGAGGGCGTGGTGTATCATACTGCAAGAATCT  Δ7-bp
GGGGTTAATTTGATTTACAAGCAGCAACCTCTTATAAGAGGGCGTGGTGTATCATACTGCAAGAATCT  WT
```

WPT312-5-56 _Dcl1b_Δ3bp _PCR.*Psi*I amplicon

```
GGGGTTGATTTGATTTACAGGCAGCAACCTCT---AAGAGGGCGTGGTGTATCATACTGCAAGAATCT  Δ3-bp
GGGGTTGATTTGATTTACAGGCAGCAACCTCT---AAGAGGGCGTGGTGTATCATACTGCAAGAATCT  Δ3-bp
GGGGTTGATTTGATTTACAGGCAGCAACCTCTTATAAGAGGGCGTGGTGTATCATACTGCAAGAATCT  WT
GGGGTTGATTTGATTTACAGGCAGCAACCTCT---AAGAGGGCGTGGTGTATCATACTGCAAGAATCT  Δ3-bp
GGGGTTGATTTGATTTACAGGCAGCAACCTCTTATAAGAGGGCGTGGTGTATCATACTGCAAGAATCT  WT
```

Status: (*dcl1a* $^{\Delta 6}$*dc1a* $^{\Delta 7}$*/DCL1b/dcl1b*$^{\Delta 3}$) without transgene

File S2. **(A)** Sequence confirmation of single *dcl1a* and *dcl1b* mutants WPT312-5- 11 d*cl1a*$^{\Delta 7}$/*dcl1a*$^{\Delta 7}$/*DCL1b*/*DCL1b* **(B)** WPT312-5-36 *dcl1a*$^{\Delta 6}$/*dcl1a*$^{\Delta 6}$/*DCL1b*/*DCL1b* **(C)** WPT312- 11 *DCL1a*/*DCL1a*/*dcl1b*$^{\Delta 15}$/*dcl1b*$^{\Delta 15}$. **(D-F)** Sequence confirmation of hemi-zygote *dcl1a*/*dcl1b* double mutants.
