# Supplementary material for: MicroRNA Maturation and MicroRNA Target Gene Expression Regulation Are Severely Disrupted in Soybean dicer-like1 Double Mutants
Source: G3 (Bethesda). 2015 Dec 15;6(2):423–33. doi: 10.1534/g3.115.022137 (PMC4751560; doi:10.1534/g3.115.022137)
Supplement: Supporting Information [file supp_g3.115.022137_FileS3.pdf]

WPT312-5-5-1

dcl1a$^{\Delta 7}$/*dcl1a*$^{\Delta 7}$/ *dcl1b*$^{\Delta 3}$/*dcl1b*$^{\Delta 3}$

A

```
GGTATGGGGTTAATTTGATTTACAAGCAGCAA-------TAAGAGGGCGTGGTGTATCATACTGCAAGAATCTTCTG   Δ7-bp
GGTATGGGGTTAATTTGATTTACAAGCAGCAA-------TAAGAGGGCGTGGTGTATCATACTGCAAGAATCTTCTG   Δ7-bp
GGTATGGGGTTAATTTGATTTACAAGCAGCAA-------TAAGAGGGCGTGGTGTATCATACTGCAAGAATCTTCTG   Δ7-bp
GGTATGGGGTTAATTTGATTTACAAGCAGCAA-------TAAGAGGGCGTGGTGTATCATACTGCAAGAATCTTCTG   Δ7-bp
GGTATGGGGTTAATTTGATTTACAAGCAGCAA-------TAAGAGGGCGTGGTGTATCATACTGCAAGAATCTTCTG   Δ7-bp
GGTATGGGGTTAATTTGATTTACAAGCAGCAA-------TAAGAGGGCGTGGTGTATCATACTGCAAGAATCTTCTG   Δ7-bp
GGTATGGGGTTAATTTGATTTACAAGCAGCAA-------TAAGAGGGCGTGGTGTATCATACTGCAAGAATCTTCTG   Δ7-bp
GGTATGGGGTTAATTTGATTTACAAGCAGCAA-------TAAGAGGGCGTGGTGTATCATACTGCAAGAATCTTCTG   Δ7-bp
GGTATGGGGTTAATTTGATTTACAAGCAGCAA-------TAAGAGGGCGTGGTGTATCATACTGCAAGAATCTTCTG   Δ7-bp
GGTATGGGGTTAATTTGATTTACAAGCAGCAACCTCTTATAAGAGGGCGTGGTGTATCATACTGCAAGAATCTTCTG   WT

AGGTATGGGGTTGATTTGATTTACAGGCAGCAACCTCT---AAGAGGGCGTGGTGTATCATACTGCAAGAATCTTCT   Δ3-bp
AGGTATGGGGTTGATTTGATTTACAGGCAGCAACCTCT---AAGAGGGCGTGGTGTATCATACTGCAAGAATCTTCT   Δ3-bp
AGGTATGGGGTTGATTTGATTTACAGGCAGCAACCTCT---AAGAGGGCGTGGTGTATCATACTGCAAGAATCTTCT   Δ3-bp
AGGTATGGGGTTGATTTGATTTACAGGCAGCAACCTCT---AAGAGGGCGTGGTGTATCATACTGCAAGAATCTTCT   Δ3-bp
AGGTATGGGGTTGATTTGATTTACAGGCAGCAACCTCT---AAGAGGGCGTGGTGTATCATACTGCAAGAATCTTCT   Δ3-bp
AGGTATGGGGTTGATTTGATTTACAGGCAGCAACCTCT---AAGAGGGCGTGGTGTATCATACTGCAAGAATCTTCT   Δ3-bp
AGGTATGGGGTTGATTTGATTTACAGGCAGCAACCTCT---AAGAGGGCGTGGTGTATCATACTGCAAGAATCTTCT   Δ3-bp
AGGTATGGGGTTGATTTGATTTACAGGCAGCAACCTCT---AAGAGGGCGTGGTGTATCATACTGCAAGAATCTTCT   Δ3-bp
AGGTATGGGGTTGATTTGATTTACAGGCAGCAACCTCT---AAGAGGGCGTGGTGTATCATACTGCAAGAATCTTCT   Δ3-bp
AGGTATGGGGTTGATTTGATTTACAGGCAGCAACCTCT---AAGAGGGCGTGGTGTATCATACTGCAAGAATCTTCT   Δ3-bp
AGGTATGGGGTTGATTTGATTTACAGGCAGCAACCTCTTATAAGAGGGCGTGGTGTATCATACTGCAAGAATCTTCT   WT
```

dcl1a$^{\Delta 7}$/*dcl1a*$^{\Delta 7}$/ *dcl1b*$^{\Delta 3}$/*dcl1b*$^{\Delta 15}$

Double mutant status of *dcl1a*$^{\Delta 7}$/*dcl1a*$^{\Delta 7}$/ *dcl1b*$^{\Delta 3}$/*dcl1b*$^{\Delta 15}$ was confirmed by PCR digest assay. This plant has not been sequence confirmed

B

M14_533

WPT312-5-5_*dcl1a*$^{\Delta 7}$*/dcl1a*$^{\Delta 7}$*/DCL1b/dcl1b*$^{\Delta 3}$*)* (female)

X

WPT312-11-1-2_*DCL1a/DCL1a/dcl1b*$^{\Delta 15}$*/dcl1b*$^{\Delta 15}$*)* *(male)*

Seed #1 from cross

M14_533-1 _ 3_*DCL1a/dcl1a*$^{\Delta 7}$*/DCL1b/dcl1b*$^{\Delta 15}$*)*

```
GGGGTTAATTTGATTTACAAGCAGCAA-------TAAGAGGGCGTGGTGTATCATACTGCAAGAATCT  Δ7-bp
GGGGTTAATTTGATTTACAAGCAGCAACCTCTTATAAGAGGGCGTGGTGTATCATACTGCAAGAATCT  WT
GGGGTTAATTTGATTTACAAGCAGCAACCTCTTATAAGAGGGCGTGGTGTATCATACTGCAAGAATCT  WT
GGGGTTAATTTGATTTACAAGCAGCAA-------TAAGAGGGCGTGGTGTATCATACTGCAAGAATCT  Δ7-bp
GGGGTTAATTTGATTTACAAGCAGCAA-------TAAGAGGGCGTGGTGTATCATACTGCAAGAATCT  Δ7-bp
GGGGTTAATTTGATTTACAAGCAGCAACCTCTTATAAGAGGGCGTGGTGTATCATACTGCAAGAATCT  WT

GGGGTTGATTTGATTTACAGGCAGCAACCTCTTATAAGAGGGCGTGGTGTATCATACTGCAAGAATCT  WT
GGGGTTGATTTGATTTACAGGCAGCAACCTCTTATA---------------TCATACTGCAAGAATCT  Δ15-bp
GGGGTTGATTTGATTTACAGGCAGCAACCTCTTATAAGAGGGCGTGGTGTATCATACTGCAAGAATCT  WT
GGGGTTGATTTGATTTACAGGCAGCAACCTCTTATA---------------TCATACTGCAAGAATCT  Δ15-bp
GGGGTTGATTTGATTTACAGGCAGCAACCTCTTATAAGAGGGCGTGGTGTATCATACTGCAAGAATCT  WT
```

Status*: (DCL1a/dc1a* $^{\Delta 7}$*/DCL1b/dc1a* $^{\Delta 15}$) without transgene

Seed #2 & 3 from cross

M14_533-2 &3_*DCL1a/dcl1a*$^{\Delta 7}$*/dcl1b*$^{\Delta 3}$*/dcl1b*$^{\Delta 15}$*)*

```
GGGGTTAATTTGATTTACAAGCAGCAA-------TAAGAGGGCGTGGTGTATCATACTGCAAGAATCT  Δ7-bp
GGGGTTAATTTGATTTACAAGCAGCAACCTCTTATAAGAGGGCGTGGTGTATCATACTGCAAGAATCT  WT
GGGGTTAATTTGATTTACAAGCAGCAACCTCTTATAAGAGGGCGTGGTGTATCATACTGCAAGAATCT  WT
GGGGTTAATTTGATTTACAAGCAGCAA-------TAAGAGGGCGTGGTGTATCATACTGCAAGAATCT  Δ7-bp
GGGGTTAATTTGATTTACAAGCAGCAA-------TAAGAGGGCGTGGTGTATCATACTGCAAGAATCT  Δ7-bp
GGGGTTAATTTGATTTACAAGCAGCAACCTCTTATAAGAGGGCGTGGTGTATCATACTGCAAGAATCT  WT

GGGGTTGATTTGATTTACAGGCAGCAACCTCT---------------TATATCATACTGCAAGAATCT  Δ7-bp
GGGGTTGATTTGATTTACAGGCAGCAACCTCT---AAGAGGGCGTGGTGTATCATACTGCAAGAATCT  Δ3-bp
GGGGTTGATTTGATTTACAGGCAGCAACCTCT---AAGAGGGCGTGGTGTATCATACTGCAAGAATCT  Δ3-bp
GGGGTTGATTTGATTTACAGGCAGCAACCTCT---------------TATATCATACTGCAAGAATCT  Δ15-bp
GGGGTTGATTTGATTTACAGGCAGCAACCTCTTATAAGAGGGCGTGGTGTATCATACTGCAAGAATCT  WT
```

Status*: (DCL1a/dc1a* $^{\Delta 7}$*/dc1b* $^{\Delta 3}$*/dc1a* $^{\Delta 15}$) without transgene

M14_534

WPT312-11-1-2_*DCL1a/DCL1a/dcl1b*$^{\Delta 15}$*/dcl1b*$^{\Delta 15}$*)*

X

WPT312-5-5_*dcl1a*$^{\Delta 7}$*/dcl1a*$^{\Delta 7}$*/DCL1b/dcl1b*$^{\Delta 3}$*)* (female)

Seed #1 & 2 from cross

M14_533-2 &3_*DCL1a/dcl1a*$^{\Delta 7}$*/dcl1b*$^{\Delta 3}$*/dcl1b*$^{\Delta 15}$*)*

```
GGGGTTAATTTGATTTACAAGCAGCAA-------TAAGAGGGCGTGGTGTATCATACTGCAAGAATCT  Δ7-bp
GGGGTTAATTTGATTTACAAGCAGCAACCTCTTATAAGAGGGCGTGGTGTATCATACTGCAAGAATCT  WT
GGGGTTAATTTGATTTACAAGCAGCAACCTCTTATAAGAGGGCGTGGTGTATCATACTGCAAGAATCT  WT
GGGGTTAATTTGATTTACAAGCAGCAA-------TAAGAGGGCGTGGTGTATCATACTGCAAGAATCT  Δ7-bp
GGGGTTAATTTGATTTACAAGCAGCAA-------TAAGAGGGCGTGGTGTATCATACTGCAAGAATCT  Δ7-bp
GGGGTTAATTTGATTTACAAGCAGCAACCTCTTATAAGAGGGCGTGGTGTATCATACTGCAAGAATCT  WT

GGGGTTGATTTGATTTACAGGCAGCAACCTCT---AAGAGGGCGTGGTGTATCATACTGCAAGAATCT  Δ3-bp
GGGGTTGATTTGATTTACAGGCAGCAACCTCT---AAGAGGGCGTGGTGTATCATACTGCAAGAATCT  Δ3-bp
GGGGTTGATTTGATTTACAGGCAGCAACCTCT---AAGAGGGCGTGGTGTATCATACTGCAAGAATCT  Δ3-bp
GGGGTTGATTTGATTTACAGGCAGCAACCTCT---------------TATATCATACTGCAAGAATCT  Δ15-bp
GGGGTTGATTTGATTTACAGGCAGCAACCTCTTATAAGAGGGCGTGGTGTATCATACTGCAAGAATCT  WT
```

Status*: (DCL1a/dc1a* $^{\Delta 7}$*/dc1b* $^{\Delta 3}$*/dc1a* $^{\Delta 15}$) without transgene

File S3. (A) Sequence confirmation of the double mutant $dcl1a^{\Delta 7}/dcl1a^{\Delta 7}/ dcl1b^{\Delta 3}/dcl1b^{\Delta 3}$. Ten clones of PCR amplicons from un-digested template were sequenced to confirm double mutant status. No wild-type alleles of Dcl1a or Dcl1b were observed. **(B)** Sequence confirmation of the M14-533 & M14-534 crosses indicating successful introgression of the $dcl1a^{\Delta 7}$ and $dcl1b^{\Delta 15}$ allele and the $dcl1a^{\Delta 7}$, $dcl1b^{\Delta 3}$ and $dcl1b^{\Delta 15}$ alleles for the development of the $dcl1a^{\Delta 7}/dcl1a^{\Delta 7}/dcl1b^{\Delta 3}/dcl1b^{\Delta 15}$ and $dcl1a^{\Delta 7}/dcl1a^{\Delta 7}/dcl1b^{\Delta 15}/dcl1b^{\Delta 15}$ double mutants.
